# Comparison of clinical characteristics and outcomes between aspiration pneumonia and community-acquired pneumonia in patients with chronic obstructive pulmonary disease

**DOI:** 10.1186/s12890-015-0064-5

**Published:** 2015-07-08

**Authors:** Yasuhiro Yamauchi, Hideo Yasunaga, Hiroki Matsui, Wakae Hasegawa, Taisuke Jo, Kazutaka Takami, Kiyohide Fushimi, Takahide Nagase

**Affiliations:** Department of Respiratory Medicine, Graduate School of Medicine, The University of Tokyo, 7-3-1 Hongo, Bunkyo-ku, Tokyo 113-8655 Japan; Division for Health Service Promotion, The University of Tokyo, 7-3-1 Hongo, Bunkyo-ku, Tokyo Japan; Department of Clinical Epidemiology and Health Economics, School of Public Health, The University of Tokyo, 7-3-1 Hongo, Bunkyo-ku, Tokyo Japan; Department of Health Policy and Informatics, Tokyo Medical and Dental University Graduate School of Medicine, 1-5-45 Yushima, Bunkyo-ku, Tokyo Japan

**Keywords:** Aspiration pneumonia, Chronic obstructive pulmonary disease, Community-acquired pneumonia, Mechanical ventilation, Mortality

## Abstract

**Background:**

Chronic obstructive pulmonary disease (COPD) patients often have dysphagia through age and several co-morbidities, leading to aspiration pneumonia (AsP). COPD patients also have increased risk of developing community-acquired pneumonia (CAP). Using a national inpatient database in Japan, we aimed to compare clinical characteristics and outcomes between AsP and CAP in COPD patients and to verify the factors that affect in-hospital morality.

**Methods:**

We retrospectively collected data on COPD patients (age ≥40 years) who were admitted for AsP or CAP in 1,165 hospitals across Japan between July 2010 and May 2013. We performed multivariable logistic regression analyses to examine the association of various factors with all-cause in-hospital mortality for AsP and CAP.

**Results:**

Of 87,330 eligible patients, AsP patients were more likely to be older, male and have poorer general condition and more severe pneumonia than those with CAP. In-hospital mortality in the AsP group was 22.7 % and 12.2 % in the CAP group. After adjustment for patient background, AsP patients had significantly higher mortality than CAP patients (adjusted odds ratio, 1.19; 95 % confidence interval, 1.08–1.32). Subgroup analyses showed higher mortality to be associated with male gender, underweight, dyspnea, physical disability, pneumonia severity, and several co-morbidities. Further, older age and worse level of consciousness were associated with higher mortality in the CAP group, whereas those were not associated in the AsP group.

**Conclusions:**

Clinical characteristics differed significantly between AsP and CAP in COPD patients. AsP patients had significantly higher mortality than those with CAP.

**Electronic supplementary material:**

The online version of this article (doi:10.1186/s12890-015-0064-5) contains supplementary material, which is available to authorized users.

## Background

Chronic obstructive pulmonary disease (COPD) is the third leading cause of death in the world [1]. COPD is characterized by persistent airflow limitation associated with chronic airway inflammation [2]. Often, exacerbation of COPD occurs in the clinical course of the disease, accelerates the rate of decline in lung function [3], and is associated with significant mortality, particularly in patients requiring hospitalization [4]. The most common cause of exacerbation appears to be respiratory tract infection; it has been reported that exacerbation is caused by bacterial infection in approximately 50 % of cases [5]. Further, COPD is one of the most frequent co-morbid conditions associated with the development of community-acquired pneumonia (CAP) [6]; COPD is the most common underlying disease in patients with CAP who require hospitalization [7], and such patients have increased mortality [8, 9]. Thus, CAP should be considered as a cause of exacerbation in the differential diagnosis and treated appropriately if present [2].

Aspiration pneumonia (AsP) is assumed to be one of the phenotypes of CAP. AsP demonstrates specific clinical features [10, 11], such as frequent occurrence in elderly patients, greater severity of pneumonia, and poorer long-term mortality compared with CAP [11–13]. COPD patients have been reported to have increased risk of dysphagia, which is related to the occurrence of AsP. This dysphagia is due to ageing [13] and also to the presence of cerebrovascular disease [11] and gastro-oesophageal reflux disease [14] as co-morbidities of COPD. COPD patients have reduced laryngopharyngeal sensitivity and impaired swallowing function compared with healthy age-matched controls [15, 16]. AsP should therefore be considered in the differential diagnosis in COPD patients with exacerbation who require hospitalization.

However, there is little information about the clinical features of COPD patients with AsP compared with those with CAP—particularly with respect to in-hospital mortality. Therefore, the purpose of this study was to compare clinical characteristics and outcomes between AsP and CAP in COPD patients using a national inpatient database in Japan. We also aimed to evaluate factors that affect in-hospital mortality in the AsP and CAP patient groups.

## Methods

### Data source

This study used the Diagnosis Procedure Combination database, a nationwide inpatient database in Japan. The database includes administrative claims data and discharge abstract data. With this database, the primary diagnosis on admission, co-morbidities present, and complications during hospitalization are recorded using the International Classification of Disease and Related Health Problems, 10th Revision (ICD-10) codes accompanied by text data in Japanese. The database also contains the following details: patient’s age, sex, body height, and weight; severity of dyspnea based on the Hugh-Jones dyspnea scale (details of dyspnea scale appear in Additional file 1: Additional Information); level of consciousness based on the Japan Coma Scale [17, 18] on admission (details of the Japan Coma Scale are shown in Additional file 1: Additional Information); grades of activities of daily life on admission converted to the Barthel index [19] (details of the Barthel index appear in Additional file 1: Additional Information); severity of pneumonia based on the A-DROP score (details of the A-DROP scoring system are shown in Additional file 1: Additional Information); admission to intensive care unit (ICU) during hospitalization; mechanical ventilation; outcome; and discharge status. Body mass index (BMI) was defined as body weight (kg) divided by the square of body height (m^2^; details of BMI categorization appear in Additional file 1: Additional Information).

This study was approved by the Institutional Review Board of The University of Tokyo. The board waived the requirement for patient informed consent because of the anonymous nature of the data.

### Patient selection

We retrospectively collected data for patients aged over 40 years who had been admitted to hospital because of AsP (ICD-10 code J69) or pneumonia caused by microbes (J10–J18) as the main diagnosis on admission, who had a diagnosis of COPD (J41–J44), and who were discharged between 1 July 2010 and 31 March 2013. To preclude hospital-acquired and ventilator-associated pneumonia, we excluded patients who had pneumonia recorded as a post-admission complication. The presence of COPD was based on physician diagnosis. We divided the patients into those with AsP and CAP.

### Co-morbidities on admission

We identified co-morbidities on admission using ICD-10 codes (detailed ICD-10 codes appear in Additional file 1: Additional Information): interstitial pneumonia; lung cancer; heart failure; ischemic heart disease; cardiac arrhythmia; pulmonary embolism; cor pulmonale; cerebrovascular disease; chronic liver disease; chronic renal failure; anxiety; depression; and bone fracture.

### A-DROP score

We evaluated the severity of pneumonia using the A-DROP score, which is a scoring system for the clinical severity of community-acquired pneumonia adopted by the Japanese Respiratory Society; it is similar to the CURB-65 of the British Thoracic Society [20]. We categorized the severity of pneumonia into four classes using the A-DROP score: mild, 0; moderate, 1–2; severe, 3; and extremely severe, 4–5.

### Clinical outcomes

The outcomes of this study were all-cause in-hospital mortality, length of hospital stay (days), length of ICU stay (days), requirement of mechanical ventilation, duration of mechanical ventilation (days), and mortality of patients who underwent mechanical ventilation.

### Statistical analysis

We used the chi-square test to compare proportions, the two-sample *t* test to compare average values, and the Mann–Whitney test to compare the median values between the groups. We performed multivariable logistic regression analyses to assess the relationship between patient-level factors and mortality in the following COPD patients—after adjustment for within-hospital clustering by means of a generalized estimating equation [21]: (1) all patients with pneumonia, including both AsP and CAP; (2) patients with AsP alone; and (3) those with CAP alone. The threshold for significance was a value of *p* < 0.05. We performed all statistical analyses using SPSS Statistics for Windows, Version 22.0 (IBM Corp., Armonk, NY, USA).

## Results

### Comparison of clinical characteristics between AsP and CAP in patients with COPD

We identified 87,330 patients with COPD (age ≥40 years) who were admitted for pneumonia; 16,388 had AsP, and 70,942 had CAP. The patient characteristics appear in Table 1. The average age in the AsP group was 82.7 years, which was significantly older than that in the CAP group (77.4 years). The average BMI in the AsP group was 18.5 kg/m^2^, which was significantly lower than that in the CAP group (19.9 kg/m^2^). The proportion of patients with severe dyspnea grade, low level of consciousness, and low Barthel index on admission was higher in the AsP group than in the CAP group; thus, the general condition on admission was poorer in the AsP group than in the CAP group. Regarding severity of pneumonia, the median A-DROP score in the AsP group was 2 (interquartile range; IQR, 2–3), which was higher than that in the CAP group (2, IQR, 1–3); the proportion of severe pneumonia was higher in the AsP group than in the CAP group. Co-morbidities on admission are presented in Table 2. The proportion of cerebrovascular disease in the AsP group was approximately 3-fold that in the CAP group. The proportion of bone fractures in the AsP group was 2-fold that in the CAP group.

**Table 1:** Patient backgrounds on admission

	AsP (n = 16,388)		CAP (n = 70,942)		*p*
Age (years) †	82.7 ± 8.0		77.4 ± 9.5		<0.001
40–64	426	(2.6)	7,318	(10.3)	
65–74	1,851	(11.3)	16,562	(23.3)	
75–84	6,935	(42.3)	30,759	(43.4)	
≥85	7,176	(43.8)	16,303	(23.0)	
Sex					<0.001
Male	13,328	(81.3)	58,815	(82.9)	
Female	3060	(18.7)	12,127	(17.1)	
Body mass index (kg/m^2^)†	18.5 ± 3.6		19.9 ± 3.8		<0.001
<18.5	7,196	(54.9)	23,835	(39.0)	
18.5–24.9	5,338	(40.7)	31,964	(52.3)	
25.0–29.9	512	(3.9)	4,684	(7.7)	
>30	58	(0.4)	631	(1.0)	
Dyspnea scale					<0.001
I	562	(3.8)	6159	(9.4)	
II	966	(6.5)	8,991	(13.7)	
III	1,239	(8.4)	9,001	(13.7)	
IV	2,509	(16.9)	16,277	(24.8)	
V	4,284	(28.9)	16,885	(25.7)	
Unspecified	5,273	(35.5)	8,348	(12.7)	
Level of consciousness					<0.001
Alert	10,233	(62.5)	60,993	(86.0)	
Dull	4,048	(24.7)	7,220	(10.2)	
Somnolence	1,379	(8.4)	1,610	(2.3)	
Coma	724	(4.4)	1,116	(1.6)	
ADL level					<0.001
20	1,633	(11.2)	23,281	(37.8)	
15–19	1,124	(7.7)	8,462	(13.7)	
10–14	1,614	(11.1)	9,887	(16.0)	
5–9	1,590	(10.9)	6,113	(9.9)	
0–4	8,598	(59.1)	13,927	(22.6)	
A-DROP score ‡	2 [2, 3]		2 [1–3]		<0.001
Mild	175	(1.9)	4,293	(8.8)	
Moderate	4,833	(53.7)	31,067	(63.6)	
Severe	2,352	(26.1)	9,630	(19.7)	
Extremely severe	1,641	(18.2)	3,868	(7.9)	

Data are expressed as numbers (%), unless otherwise indicated; † data are expressed as averages ± standard deviation; ‡ data are expressed as medians [interquartile range]. *Abbreviations:* *AsP* aspiration pneumonia, *CAP* community-acquired pneumonia, *ADL* activities of daily life

**Table 2:** Co-morbidities on admission

	AsP		CAP		*p*
	n = 16,388	(%)	n = 70,942	(%)	
Interstitial pneumonia	357	(2.2)	3,619	(5.1)	<0.001
Lung cancer	728	(4.4)	6,983	(9.8)	<0.001
Heart failure	3,350	(20.4)	12,704	(17.9)	<0.001
Ischemic heart disease	998	(6.1)	4,619	(6.5)	0.048
Cardiac arrhythmia	880	(5.4)	3,560	(5.0)	0.065
Pulmonary embolism	31	(0.2)	206	(0.3)	0.025
Cor pulmonale	110	(0.7)	806	(1.1)	<0.001
Cerebrovascular disease	2,816	(17.2)	3,992	(5.6)	<0.001
Chronic liver disease	171	(1.0)	1,102	(1.6)	<0.001
Chronic renal failure	345	(2.1)	1,525	(2.1)	0.723
Anxiety/depression	227	(1.4)	1,025	(1.4)	0.562
Bone fracture	353	(2.2)	812	(1.1)	<0.001

Data are expressed as numbers (%) in each category. *Abbreviations:* *AsP* aspiration pneumonia, *CAP* community-acquired pneumonia

### Comparison of outcomes between AsP and CAP in patients with COPD

The outcomes appear in Table 3. All-cause in-hospital mortality in the AsP group was 22.7 %, whereas it was 12.2 % in the CAP group. Hospital stay in the AsP group was longer than in the CAP group. The proportions of ICU admission and requirement of mechanical ventilation in the AsP group were higher than in the CAP group. The length of ICU stay was similar in the two groups. The mortality rate in patients who required mechanical ventilation in the AsP group was 51.8 % (n = 1,010/1,951), which was significantly higher than in the CAP group (41.4 %, n = 3,036/7,339).

**Table 3:** Clinical outcomes

		AsP (n = 16,388)		CAP (n = 70,942)		*p*
Death		3,726	(22.7)	8,671	(12.2)	<0.001
Length of stay (days)		21.0 [12–40]		15.0 [10–26]		<0.001
ICU admission		522	(3.2)	2021	(2.8)	0.021
Length of ICU stay (days)		4.0 [2–10]		4.0 [2–9]		0.527
Mechanical ventilation		1,951	(11.9)	7,339	(10.3)	<0.001
Duration of mechanical ventilation (days)		6.0 [1–21]		7.0 [2–20]		0.001
	Death†	1,010	(51.8)	3,036	(41.4)	<0.001

Data are expressed as numbers (%) or medians [interquartile range];†% indicates the proportion of patients who died, among those who underwent mechanical ventilation. *Abbreviations*: *AsP* aspiration pneumonia, *CAP* community-acquired pneumonia, *ICU* intensive care unit

### Multivariable logistic regression analysis for all-cause in-hospital mortality

The results of the multivariable logistic regression analysis for all-cause in-hospital mortality are presented in Table 4. In the analysis for all patients (left-hand column, Table 4), AsP was significantly associated with higher mortality (adjusted odds ratio, 1.19; 95 % confidence interval, 1.08–1.31; *p* = 0.001) than CAP. Higher mortality was associated with older age, male gender, lower BMI, more severe dyspnea scale, worse level of consciousness, worse level of activities of daily life, and more severe pneumonia. Higher mortality was also associated with several co-morbidities on admission, including interstitial pneumonia, lung cancer, heart failure, cor pulmonale, chronic renal failure, and bone fracture.

**Table 4:** Multivariable logistic regression analysis for in-hospital death

		All		AsP		CAP	
		aOR	95 % CI	aOR	95 % CI	aOR	95 % CI
Type of pneumonia							
	AsP	1.19**	1.08–1.31				
	CAP	Ref.					
Age (years)							
	40–64	Ref.		Ref.		Ref.	
	65–74	1.13	0.93–1.38	1.43	0.75–2.71	1.09	0.87–1.35
	75–84	1.28*	1.05–1.56	1.52	0.80–2.91	1.22	0.98–1.52
	>85	1.45**	1.18–1.77	1.58	0.83–2.97	1.41**	1.12–1.77
Sex							
	Male	Ref.		Ref.		Ref.	
	Female	0.60**	0.54–0.67	0.66**	0.52–0.83	0.58**	0.52–0.66
Body mass index (kg/m^2^)							
	<18.5	1.70**	1.58–1.83	1.72**	1.50–1.96	1.69**	1.55–1.84
	18.5–24.9	Ref.		Ref.		Ref.	
	25–29.9	0.77**	0.65–0.93	0.73	0.48–1.12	0.77*	0.63–0.95
	>30	0.88	0.56–1.38	0.79	0.24–2.55	0.90	0.52–1.47
Dyspnea grade by Hugh-Jones classification							
	I	Ref.		Ref.		Ref.	
	II	1.23	0.89–1.68	0.64	0.33–1.22	1.42	1.00–2.02
	III	1.51**	1.13–2.01	0.90	0.51–1.58	1.68**	1.21–2.34
	IV	2.26**	1.74–2.96	1.30	0.76–2.22	2.57**	1.89–3.50
	V	5.44**	4.19–7.08	2.60**	1.55–4.37	6.42**	4.72–8.72
	Unspecified	5.54**	4.20–7.29	2.47**	1.46–4.19	6.90**	4.99–9.53
Level of consciousness							
	Alert	Ref.		Ref.		Ref.	
	Dull	0.96	0.86–1.07	0.88	0.75–1.03	1.00	0.88–1.14
	Somnolence	1.09	0.94–1.27	0.82	0.64–1.04	1.38**	1.12–1.70
	Coma	1.46**	1.19–1.80	1.22	0.88–1.68	1.62**	1.24–2.10
Level of ADL scored by Barthel index							
	20	Ref.		Ref.		Ref.	
	15–19	1.26**	1.09–1.45	1.07	0.73–1.59	1.26**	1.07–1.48
	10–14	1.54**	1.34–1.76	1.33	0.93–1.90	1.51**	1.31–1.75
	5–9	1.90**	1.65–2.20	1.22	0.85–1.75	1.99**	1.69–2.35
	0–4	3.04**	2.67–3.46	1.94**	1.43–2.63	3.21**	2.76–3.71
A-DROP score							
	Mild	Ref.		Ref.		Ref.	
	Moderate	1.64**	1.24–2.17	3.22*	1.15–9.00	1.48*	1.09–2.01
	Severe	2.86**	2.12–3.85	5.11**	1.78–14.65	2.67**	1.94–3.68
	Extremely severe	7.84**	5.74–10.70	14.18**	4.85–41.41	7.22**	5.15–10.12
Co-morbidities on admission							
	IP	2.21**	1.88–2.60	1.69*	1.12–2.55	2.32**	1.95–2.76
	Lung cancer	2.80**	2.45–3.20	1.88**	1.40–2.52	3.00**	2.59–3.48
	Heart failure	1.16**	1.06–1.27	1.27**	1.06–1.53	1.14*	1.00–1.24
	IHD	0.80**	0.69–0.93	0.93	0.69–1.25	0.76**	0.63–0.90
	Arrhythmia	1.15	0.99–1.33	1.34*	1.01–1.78	1.07	8.9–.128
	PE	0.91	0.44–1.90	1.20	0.11–12.56	0.84	0.40–1.75
	Cor pulmonale	1.42*	1.02–1.99	1.77	0.80–3.89	1.35	0.93–1.l97
	CVD	0.94	0.84–1.06	0.99	0.84–1.16	0.92	0.79–1.07
	CLD	1.23	0.93–1.64	1.51	0.81–2.81	1.13	0.81–1.57
	CRF	1.65**	1.35–2.02	1.56*	1.08–2.27	1.68**	1.35–2.09
	ANX/DEP	0.90	0.64–1.28	1.53	0.92–2.53	0.67	0.43–1.05
	Bone fracture	1.66**	1.29–2.13	1.20	0.80–1.82	1.90**	1.41–2.56

*p value <0.05, **p value <0.01. *Abbreviations*: *AsP* aspiration pneumonia, *CAP* community-acquired pneumonia, *aOR* adjusted odds ratio, *CI* confidence interval, *Ref*. reference, *ADL* activities of daily life, *IP* interstitial pneumonitis *IHD* ischemic heart disease, *PE* pulmonary embolism, *CVD* cerebrovascular disease, *CLD* chronic liver disease, *CRF* chronic renal failure, *ANX/DEP* anxiety and depression

Subgroup analyses of the AsP and CAP groups (center and right-hand columns, Table 4) showed that higher mortality was commonly associated in both groups with male gender, underweight, dyspnea, physical disability, severe pneumonia, interstitial pneumonitis, lung cancer, heart failure, and chronic renal failure. Further, older age and worse level of consciousness were associated with higher mortality in the CAP group, whereas those were not associated with mortality in the AsP group.

## Discussion

Using a national inpatient database in Japan, we compared clinical characteristics and outcomes between AsP and CAP in patients with COPD. The AsP group was more likely to be older, male, have lower BMI, more severe dyspnea, worse levels of consciousness, worse activities of daily life level, and greater severity of pneumonia than the CAP group. Mortality in the AsP group was significantly higher than in the CAP group—even after adjustment for patient backgrounds.

It is well known that there is a high incidence of aspiration in the elderly, and it is an important risk factor in CAP [22]. One study has reported the incidence of aspiration in elderly CAP patients to be 71 %, which was significantly higher than control subjects without acute disease (10 %) [23]. In addition, COPD patients have been found to have a high incidence of impairment of the swallowing reflex, which is a risk factor for exacerbation of COPD [14, 24] and is related to the presence of gastro-oesophageal reflux disease as a co-morbidity [14, 25]. Further, COPD patients have increased risk of mild cognitive impairment [26], which is associated with swallowing difficulties [27]. Moreover, patients with COPD have impaired laryngopharyngeal sensitivity, which leads to inability to detect pharyngeal residue and subsequent aspiration of pharyngeal contents [15]. Thus, COPD patients have increased risk of AsP.

AsP is believed to be one of the phenotypes of CAP. Although there are no definitive diagnostic criteria of AsP, it is generally characterized by aspiration of oropharyngeal contents into the larynx and lower respiratory tract [10, 28]. Previous studies have demonstrated that AsP has distinct clinical characteristics: compared with CAP, it appears at an older age in individuals with lower BMI and is associated with greater severity of pneumonia and increased long-term mortality [11, 12]. The present study found the clinical characteristics on admission in the AsP group to be similar to those reported in previous investigations [11, 12], and AsP in COPD patients also has distinct clinical features.

Regarding mortality, it is controversial whether or not the presence of AsP in the general population is an independent short-term prognostic factor. After adjusting for co-morbidities and severity of pneumonia, some studies [11, 12] have found that AsP was not associated with short-term mortality. Adopting a strict diagnosis for AsP, another investigation [29] has demonstrated that AsP is an independent prognostic factor for short-term mortality in pneumonia patients. Our search of the medical literature revealed no report that focused on AsP mortality in COPD patients. The present study showed AsP in COPD patients to be associated with higher short-term mortality—even after adjustment for patient backgrounds. The association between AsP in COPD and mortality could be attributed to the following: AsP patients easily develop recurrent pneumonia [11], which causes a deterioration in lung function in COPD patients [3]; because COPD patients have increased risk of aspiration, COPD patients with AsP have more repetitive aspiration events—even during hospitalization [29]. Thus, AsP in COPD patients would appear to be associated with higher mortality.

The length of hospital stay in the AsP group was significantly greater than in the CAP group, which is consistent with the results of previous studies that focused on AsP. [11, 12, 30] Likewise, the proportions of ICU admission and requirement of mechanical ventilation were higher in the AsP than in the CAP group, which is a similar finding to that reported elsewhere [11, 30]. To our knowledge, no previous study has examined AsP mortality in COPD patients who require mechanical ventilation. One investigation on mortality in CAP patients undergoing mechanical ventilation found an overall mortality rate of 32 % [31]; the mortality in the AsP group in the present study was 51.8 %, which is much higher than the earlier figure.

This study has several limitations. The diagnoses of COPD, AsP, and CAP were based on physician-based assessments. The accuracy of the diagnosis was not certified by specialists. However, in normal clinical settings, these diseases are not always diagnosed by specialists, and so we believe the data relating to physician-based diagnosis to be meaningful. In addition, cases of pneumonia not having been diagnosed on admission but being diagnosed within the first 48 hours after admission may have been excluded in this study: such cases may not have appeared in the category of hospital-acquired pneumonia. Thus, AsP and CAP may have been underestimated in the present study. Further, the degree of airflow limitation was not evaluated in this study: the database does not include the stages of COPD severity or details of pulmonary function tests, including forced expiratory volume in 1 second and other indices. Thus, we cannot evaluate the association between mortality and degree of lung function.

## Conclusion

In conclusion, this study demonstrates that AsP has different characteristics to CAP and that AsP is associated with higher in-hospital mortality than CAP in COPD patients.

## Additional file

Additional file 1:
**Additional Information.**

## Abbreviations

COPD: Chronic obstructive pulmonary disease
CAP: Community-acquired pneumonia
AsP: Aspiration pneumonia
ICD-10: International classification of disease and related health problems 10th revision
ICU: Intensive care unit
BMI: Body mass index
