# Supplementary material for: Comparison of clinical characteristics and outcomes between aspiration pneumonia and community-acquired pneumonia in patients with chronic obstructive pulmonary disease
Source: BMC Pulm Med. 2015 Jul 8;15:69. doi: 10.1186/s12890-015-0064-5 (PMC4495636; doi:10.1186/s12890-015-0064-5)
Supplement: Additional file 1: — Additional Information. [file 12890_2015_64_MOESM1_ESM.docx]

Additional Information

**Methods**

*Grading of dyspnea severity*

Based on the Hugh-Jones classification,[[1](#_ENREF_1)] the grading of dyspnea severity was defined as follows: (I) the patient’s breathing was as good as that of other people their age and build when working, walking, and climbing hills or stairs; (II) the patient was able to walk at the pace of normal people of their age and build on level ground but was unable to maintain that pace when climbing hills or stairs; (III) the patient was unable to keep up with normal people of their age and build on level ground but was able to walk about 1.6 km or more at their own speed; (IV) the patient was unable to walk more than about 50 m on level ground without a rest; (V) the patient was breathless when talking or undressing or was unable to leave home because of breathlessness; (unspecified) the patient was unable to be classified into the above grades because of their bedridden status.

*Levels of consciousness*

Level of consciousness on the Japan Coma Scale[[2](#_ENREF_2), [3](#_ENREF_3)] was defined as follows: one-digit codes (1–3) were given to patients who were conscious without any stimuli; two-digit codes (10–30) were assigned to patients who could be aroused by some stimuli; three-digit codes (100–300) were given to patients in a coma. The Japan Coma Scale and Glasgow Coma Scale assessments are well correlated.[[4](#_ENREF_4)]

*Grading activities of daily life*

Grading of activities of daily life was performed using the Barthel index,[[5](#_ENREF_5)] which assesses functional status and ability in performing daily activities. The Barthel index consists of 10 factors: feeding, bathing, grooming, dressing, bowels, bladder, toilet use, transfer, mobility, and stairs. Scores range from 0 to 20, with a score of 20 indicating total independence, lower scores indicating increasing dependence, and 0 signifying complete dependence.[[6](#_ENREF_6)] It should be noted that changes of more than two points (10%) in the total score accurately reflect changes in functional status.[[7](#_ENREF_7)] We categorized patients into five groups according to the score: totally independent (20); partially independent; (19–15, 14–10, 9–5); and dependent (4–0).

*Body mass index categories*

The body mass index categories were assigned based on World Health Organization classifications of underweight (<18.5 kg/m^2^), normal weight (18.5–24.9 kg/m^2^), overweight (25.0–29.9 kg/m^2^), and obese (≥30.0 kg/m^2^) individuals.[[8](#_ENREF_8)]

*A-DROP scores*

The severity of pneumonia was evaluated with the A-DROP score, which uses a six-point scale (0–5) to assess the clinical severity of community-acquired pneumonia; it was established by the Japanese Respiratory Society and is reportedly similar to the CURB-65 of the British Thoracic Society.[[9](#_ENREF_9)] The A-DROP score consists of the following parameters: (1) age (male ≥70 years, female ≥75 years); (2) dehydration (blood urea nitrogen (BUN) ≥21 mg/dL); (3) respiratory failure (SaO_2_ ≤90% or PaO_2_ ≤60 mmHg); (4) orientation disturbance (confusion); and (v) low blood pressure (systolic blood pressure ≤90 mmHg). The A-DROP score is a modified version of the CURB-65: confusion; BUN ≥7 mmol/L (19 mg/dL); respiratory rate ≥30/min; low blood pressure (diastolic ≤60 mmHg or systolic <90 mmHg); and age ≥65 years. The severity of pneumonia was classified into four categories using the A-DROP score: mild (0); moderate (1–2); severe (3); and extremely severe (4–5).

*ICD-10 codes of co-morbidities*

Co-morbidities on admission were identified using ICD-10 codes: interstitial pneumonia (J84); lung cancer (C34); heart failure (I50); ischemic heart disease (I20–I22, I25); cardiac arrhythmia (I44–I45, I47–I49); pulmonary embolism (I26); cor pulmonale (I27); cerebrovascular disease (I60–I69); chronic liver disease (K70–71, K73–74, K76); chronic renal failure (N18); anxiety (F40–41); depression (F30–33); and bone fracture (S02, S12, S22, S32, S42, S52, S62, S72, S82, S92, T02, T10, T12).

**References**

1. Hugh-Jones P, Lambert AV: **A simple standard exercise test and its use for measuring exertion dyspnoea.** *Br Med J* 1952, **1**:65-71.

2. Ohta T, Waga S, Handa W, Saito I, Takeuchi K: **[New grading of level of disordered consiousness (author's transl)].** *No Shinkei Geka* 1974, **2**:623-627.

3. Todo T, Usui M, Takakura K: **Treatment of severe intraventricular hemorrhage by intraventricular infusion of urokinase.** *J Neurosurg* 1991, **74**:81-86.

4. Ono K, Wada K, Takahara T, Shirotani T: **Indications for computed tomography in patients with mild head injury.** *Neurol Med Chir (Tokyo)* 2007, **47**:291-297; discussion 297-298.

5. Mahoney FI, Barthel DW: **FUNCTIONAL EVALUATION: THE BARTHEL INDEX.** *Md State Med J* 1965, **14**:61-65.

6. Collin C, Wade DT, Davies S, Horne V: **The Barthel ADL Index: a reliability study.** *Int Disabil Stud* 1988, **10**:61-63.

7. Giang P, Williams A, Argyros L: **Automated extraction of the barthel index from clinical texts.** *AMIA Annu Symp Proc* 2013, **16**:486-495.

8. World Health Organization, **Glabal Database on Body Mass Index** [[www.who.int/bmi/index.jsp?introPage=intro_3.html](http://www.who.int/bmi/index.jsp?introPage=intro_3.html)] Accessed October 26 2014.

9. Shindo Y, Sato S, Maruyama E, Ohashi T, Ogawa M, Imaizumi K, Hasegawa Y: **Comparison of severity scoring systems A-DROP and CURB-65 for community-acquired pneumonia.** *Respirology* 2008, **13**:731-735.
